# Comparison of the effect of mint extract and chamomile drops on the gastric residual volume of traumatic patients under mechanical ventilation and nasogastric tube feeding in the intensive care unit: A triple -blind, randomized, crossover trial

**DOI:** 10.22038/AJP.2023.21889

**Published:** 2023

**Authors:** Abbasali Ebrahimian, Samaneh Rahbar, Setareh Homami, Fatemeh Paknazar, Ali Fakhr-Movahedi

**Affiliations:** 1 *Department of Health in Emergencies and Disasters, Faculty of Paramedical, Qom University of Medical Sciences, Qom, Iran*; 2 *Student Research Committee, School of Nursing, Semnan University of Medical Sciences, Semnan, Iran*; 3 *Department of Midwifery, School of Nursing and Midwifery, Semnan University of Medical Sciences, Semnan, Iran*; 4 *Social Determinants of Health Research Center, Semnan University of Medical Sciences, Semnan, Iran*; 5 *Nursing Care Research Center, Semnan University of Medical Sciences, Semnan, Iran*; 6 *Department of Pediatric and Neonatal Nursing, School of Nursing, Semnan University of Medical Sciences, Semnan, Iran*

**Keywords:** Alternative medicine, Mint extract, Chamomile, Gastric residual volume, Trauma

## Abstract

**Objective:**

Mint and chamomile can effectively reduce the gastric residual volume (GRV). This study aimed to determine the effect of mint extract and chamomile drops on the GRV of trauma patients under mechanical ventilation and nasogastric tube feeding in the intensive care unit.

**Materials and Methods:**

This study was a triple-blinded randomized clinical trial with a 2×2 crossover design. Eighty patients were randomly divided to receive mint extract and chamomile drops. Five drops of mint extract and 11 drops of chamomile were gavaged every 6 hr. GRV was measured using a syringe-aspiration method before and 3 hr after each intervention. After a 24-hour washout period, the two groups changed places.

**Results:**

In the first phase of the study, before the interventions, the GRV in the mint and chamomile groups was 14.60±7.89 and 13.79±7.12 ml, and after the interventions were 8.13±6.31 and 6.61±4.68 ml, respectively. In the study's second phase, before the interventions, the GRV in the mint and chamomile groups was 10.03±4.93 and 11.46±7.17 ml and after the interventions, GRV was 4.97±4.05 and 6.98±4.60 ml, respectively. The difference in the GRV before and after the intervention was not significantly different between the two groups. Both herbal drugs effectively reduced the GRV (p=0.382).

**Conclusion:**

Mint extract and chamomile drops are similarly effective in reducing the GRV in trauma patients under mechanical ventilation and nasogastric tube (NGT) feeding in the intensive care unit.

## Introduction

Nutritional support is one of the critical aspects of providing care and treatment to patients hospitalized in intensive care units (ICUs) (Shahriari et al., 2015 ▶), and it is usually performed through a nasogastric tube after stabilizing the patient's hemodynamic condition (Aslani et al., 2015 ▶). Despite various benefits, it has several complications, the most important of which are delayed gastric discharge, increased gastric residual volume (GRV) (with a prevalence of 39%), and nasogastric intubation intolerance (with a prevalence of 30-51%) (Farsi et al., 2020 ▶; Hekmatafshar et al., 2012 ▶). Some studies have reported no association between Ventilator-associated pneumonia (VAP) and GRV and argued that GRV monitoring is unnecessary for ICU patients (Faramarzi et al., 2020 ▶; Wang et al., 2019 ▶). Marshall Smith (2020) ▶ showed that removing GRV routine assessment is safe and improves nutrition (Smith, 2020 ▶). However, others reported an increase in GRV and gastric reflux in patients with mechanical ventilation (up to 50%) (Nguyen et al., 2008 ▶). The delayed gastric discharge causes serious complications such as pneumonia and malnutrition (Nassaji et al., 2010 ▶; Vahdat Shariatpanahi et al., 2009 ▶). The prevalence of lung gastric content aspiration in mechanically-ventilation patients feeding with the nasal-gastric tube is reported to be up to 95% (Hekmatafshar et al., 2012 ▶).

To reduce gastrointestinal complications caused by GRV monitoring in patients undergoing ventilation, ICU nurses use methods to facilitate gastric discharge so that GRV monitoring is not needed anymore (Wiese et al., 2020 ▶). Several measures are developed to facilitate gastric clearance, including placing patients in Semi-recumbent (SR), Right Lateral positions (RL), and Prone positions (Farsi et al., 2020 ▶; Machado et al., 2020 ▶), abdominal massage (El-Feky, 2020 ▶), jejunostomy feeding (Ranjithatharsini, 2014 ▶), and using prokinetic drugs (e.g., metoclopramide) (Camilleri et al., 2013 ▶). However, using these methods is difficult and time-consuming and, in some exceptional cases, may cause other complications. Therefore, using traditional medicine and complementary approaches can be helpful, along with administering these medications.

Historically, medicinal plants are considered critical therapeutic measures in traditional medicine. Medicinal plants are safe, cheap, and available therapeutic options with fewer side effects. Hence, it can be used as an alternative to chemical drugs with frequent complications (Ataollahi and AliAkbari, 2014 ▶). Two commonly used plants to treat gastrointestinal problems in traditional medicine are mint and chamomile.

Mint (scientifically known as Mentha piperita L.) is from the Lamiaceae family and is an aromatic and appetite stimulant plant that has been used to treat gastrointestinal disorders. Mint has antimicrobial, anticancer, anti-cough, and anti-asthma properties and helps food digestion by stimulating gastrointestinal glands and increasing sedation and enzyme discharge, and reduces common gastrointestinal problems such as heartburn, nausea, cough, cramps, and colon spasms (Mamadalieva et al., 2017 ▶; Tandan Neeraj, 2013 ▶).

Chamomile (scientifically known as Matricaria chamomilla L.) is another medical plant from Asteraceae that is used in traditional medicine as a sedative, antispasmodic, and anti-inflammatory agent and for treatment of skin diseases (e.g. psoriasis and eczema), bronchitis, colds, cough, and inflammation, wound healing, and treatment of gastrointestinal problems. This plant extract is also used to treat gastrointestinal disorders and gastric ulcers because of its anti-lysis and anti-spasm characteristics (Ranjbar et al., 2015 ▶; Vahidi, 2000 ▶; Wuthrich and Rapee, 2013 ▶). Chamomile has antinausea and stomach sedative effects and is helpful for nerve prophesied and relaxation in stressful situations (Modareset al., 2012 ▶). This herbal medicine is available in the pharmacopeia of 26 countries (Singh et al., 2011 ▶).

Researchers found no study on the effects of mint extract or chamomile on GRV. However, these two plants have improved some other digestive disorders. Shaheenfar et al. used the mint extract to treat nausea and vomiting after a c-section and showed that mint could prevent nausea and vomiting in women who had c-sections (Sahhinfar et al., 2017 ▶). Amzajerdi et al. also showed that mint extract effectively reduced nausea (Amzajerdi et al., 2019 ▶). It seems that increasing the rate of gastric discharge can decrease nausea and vomiting in these patients.

Jabri et al. reported that chamomile could modulate the movements of the stomach and intestine and intestinal transmission of water and electrolyte. Besides, having high amounts of active biological compounds, it can prevent gastrointestinal diseases and maintain intestinal comfort (Jabri et al., 2020 ▶). In contrast, Bradley reported that chamomile could increase the rate of food discharge from the stomach by increasing gastric pH (Bradley, 2017 ▶).

Therefore, some studies have shown that nurses tend to change evidence on the GRV monitoring process (Ozen et al., 2018 ▶), and measuring and increasing GRV is a challenge for ICU staff and preventing increased GRV in patients fed with a gastric nasal tube should be prioritized (Bloomer et al., 2017 ▶; Modarres et al., 2011 ▶; Smith, 2020 ▶). Since studies do not support the effect of extracts of mint and chamomile on increasing the rate of gastric discharge, the current study aimed to compare these extracts on GRV in patients fed with the nasal-gastric tube in the intensive care unit.

## Materials and Methods

**Study design and participants**

**Figure 1:**
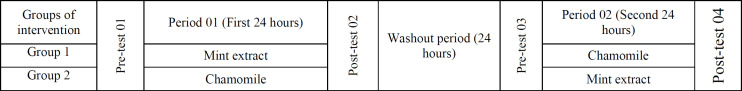
Design of the study

This study was a triple-blinded randomized clinical trial (ID: IRCT20151020024625N10) with a 2×2 crossover design conducted between January 10, 2019, and August 31, 2019. In this study, the patient did not know what solution was used for him/her. The nurse who did the sampling did not know that the solution she was gavaging for the patient was mint extract or chamomile drops, and the statistic’s specialist did not know which groups received mint extract and which groups received chamomile drops. In this study, the crossover method was used to control all sub-confounders. Each sample received both interventions in this method and is considered its control. To perform the crossover method, the study was designed in two phases. In the first phase, the samples were randomly divided into two groups: Mint and Chamomile. For patients in the Mint group, 40 drops of mint extract, and for patients in the Chamomile group, 90 drops of chamomile were gavaged every 6 hr, and the first phase ended after 24 hr. Then, we stopped the interventions in both groups for 24 hr to accomplish washout and remove the carryover effect. In the second phase, the groups were replaced. Patients who received mint extract in the first phase received chamomile drops in the second phase, and patients who received chamomile drops in the first phase received mint extract in the second phase. The second phase lasted 24 hr. Patients received medication through the nasogastric tube every 6 hr (Figure 1).

The study population comprised 80 patients who underwent nasogastric tube nutrition in the trauma ICU of Kowsar hospital in Semnan, Iran. The study sample consisted of those patients who met the following inclusion criteria: stable hemodynamic conditions, no opium drug use in his/her history, no history of allergy to mint extract or chamomile drops, feeding through the Naso-Gastric Tube (NGT), and being under mechanical ventilation with supportive modes.

The exclusion criteria comprised of unwillingness to participate in the study, sensitivity to mint extract or chamomile drops, any allergic reaction during the study, any emergency requiring immediate intervention, the occurrence of nausea and vomiting during the study, an GRV more than 50 ml, receiving physician's order to stop feeding through the NGT and physician's order to remove the NGT.

**Sample size**

The sample size was estimated based on a pilot study conducted on 20 patients in two groups (each with ten patients). The sample size was calculated using the PASS 21 software with a significance level of 5%, a test power of 90%, and the mean difference between the mint extract and chamomile groups was 4.61±0.52. To determine the sample size, the “Tests for the difference between two means in a 2×2 crossover design method” were used, and the sample size was obtained as 38 patients per group. The probability of dropping samples was considered in this study, so 40 patients were considered for each group.

**Measures**

The data collection tools were demographic and physiological parameter 

information and a gastric residual volume (GRV) sheet. The data collection tools were completed after obtaining informed consent. This information includes age, gender, underlying diseases such as hypertension, diabetes mellitus, chronic and obstructive pulmonary disease (COPD), blood pressure, heart rate, temperature, and spontaneously respiratory rate.

**Randomization**

In the first phase of the study, the patients who underwent NGT nutrition and met the inclusion criteria were randomly divided into the Mint extract group (A group) and the Chamomile drops group (B group). Computerized block randomization was used for random allocation. This block was AABB, ABAB, BAAB, BABA, ABBA, BBAA, ABAB, ABBA, AABB and BABA. Therefore, the first and second patients in the Mint group, the second and third patients in the Chamomile group, the fifth in the Mint group, the sixth in the Chamomile group, and so on were included. In cases where a participant was excluded from the study, the first patient admitted to the ward who met the inclusion criteria was replaced. 

**Intervention**

This study was performed with a 2×2 crossover design. Before sampling, forty drops of mint extract solution 20% (Barij Essence Co., Kashan, Iran) and 90 drops of chamomile solution (Zardband Co., Tabriz, Iran) were separately cast into two previously sterilized glass containers. Then, sterile distilled water was added, and the volume of the solution was increased to 40 ml. The prepared solutions were placed in the refrigerator, and solutions were labelled as A (mint extract) and B (chamomile). The nurse performing the sampling was blinded about the type of solutions. The manufacturer's recommendations for these two herbal medicines determined the dosage of mint extract and chamomile drops.

According to the routine activities of the ward, nasogastric gavage feeding was performed every 3 hr (Hours 9, 12, 15, 18, 21, 24, 3, and 6) (Premji and Chessell, 2011 ▶). However, we performed the interventions in both groups every 6 hr (Hours 9, 15, 21, and 3). Before nasogastric gavage feeding, the nurse placed the patients in a semi-sitting position with a 30°angle. Then, the GRV was measured and recorded using the syringe-aspiration technique. To observe ethics, if GRV was more than 50 ml, the patient was excluded because the safe volume of GRV for patients is 50 ml (Johnson, 2009 ▶). According to the obtained number, the patient's GRV was decreased from 300 ml, and he/she was prepared for nasogastric gavage feeding. The nasogastric gavage feeding was gently performed in 10 min using a 50 ml gavage injector. In the study's first phase, the nasogastric gavage feeding of A (mint extract) and B (chamomile) solutions was performed randomly. Then, to ensure the complete entrance of the drugs into the stomach, 20 ml water was gavaged, and NGT was clamped. To determine the effect of the interventions, 3 hr after the food fluid gavage through that NGT (Hours 12, 18, 24, and 6) and before the next stage of gastric food fluid gavage, the patients' GRV was measured by using the syringe-aspiration technique and recorded. 

Interventions were stopped for one day in both groups (Washout period). In the second phase of the study, the positions of the two groups were changed, meaning that solution B was gavaged to the first group and solution A was gavaged to the second group (Figure 1). To determine the effectiveness of each intervention, the average of 6-hr measurements before and 3 hr after each test was calculated. The therapeutic effect (The primary out com) was calculated based on the mean difference before and after mint extract and chamomile interventions in the two sequences. 

**Statistical methods**

The data were analysed using descriptive statistics, including frequency, mean, and standard deviation. The Chi-square test was applied to compare the patients' gender and underlying diseases. 

The independent sample t-test was applied to compare the patients' mean age, blood pressure, heart rate, temperature, spontaneously respiratory rate, and gastric residual volume between the two groups before the interventions.

The analysis of 2×2 crossover designs using the T-test was performed to compare the patients’ mean gastric residual volumes between the two groups. The paired-sample T-test was performed to compare the mean gastric residual volumes before and after each intervention in the two groups. All the analyses were conducted in NCSS 21 and SPSS-26. The level of statistical significance was set at p<0.05 for all statistical analyses.

**Ethical considerations**

This study protocol was approved by the Ethics Committee of Semnan University of Medical Sciences (approval code: IR.SEMUMS.REC.1397.219) and registered at the Iranian Registry of Clinical Trials (registration code: IRCT20151020024625N10). All patients/patient families participating in the study, signed informed consent form before sampling. The study objectives and procedure were also explained to the patients/patient's family, and informed consent was obtained.

## Results

**Figure 2:**
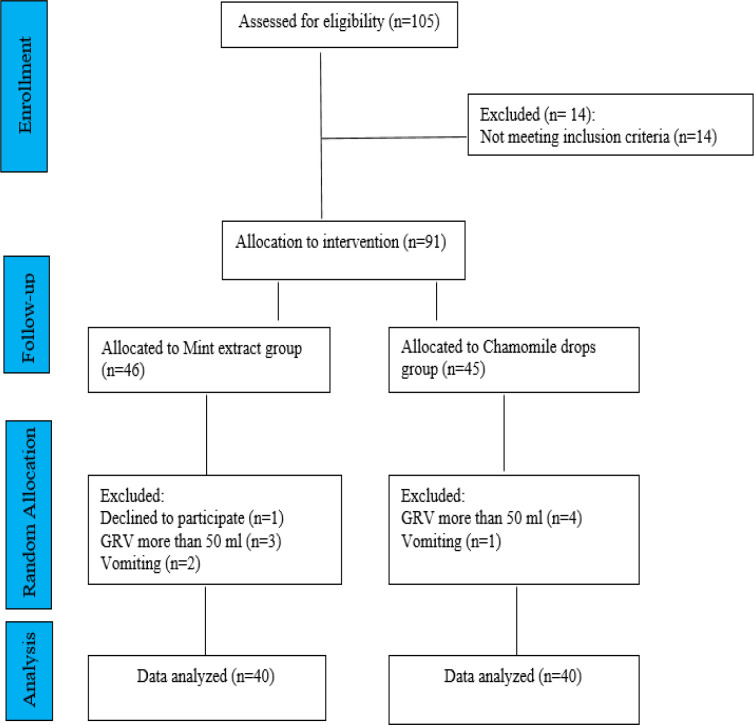
CONSORT flowchart of the study

A total of 105 patients underwent NGT nutrition in Kowsar trauma ICU during the sampling process; 91 patients met the inclusion criteria. Eleven patients were excluded from the study during the sampling process because of unwillingness to participate in the study (one patient), vomiting (three patients), a GRV more than 50 ml (seven patients), and finally, the data of 80 patients were analyzed (Figure 2). The participants were 49 males (61.25%) and 31 females (38.75%). Their mean age was 58.59±21.90 years. Twelve patients (15%) had a history of hypertension, 3 (3.75%) had diabetes, 1 (1.25%) had COPD, and 35 (43.75%) had more than one type of underlying diseases. There was no significant difference in individual characteristics (age, gender, type of underlying disease, gastric residual volume, blood pressure, heart rate, spontaneously respiratory rate, and temperature) between the two groups before the intervention, patients in both groups were matched in terms of individual characteristics (p>0.05) (Table 1). The amounts of GRV before the interventions (Hours 9, 15, 21, and 3) were considered the baseline, and the amounts of GRV 3 hr after the interventions (hours 12, 18, 24, and 6) were considered the values indicating the effect of interventions in both groups. Also, the difference between the baseline GRV and 3 hr later was calculated and used in statistical analysis (Table 2). Paired-sample T-test revealed a statistically significant difference in the means of GRV before and after the interventions in both groups (p<0.05) (Table 2).

Therefore, mint extract and chamomile drops effectively reduced the GRV of patients fed through the NGT. Analysis of 2x2 crossover designs using the T-test showed that both mint extract and chamomile drop reduced the GRV in the patients equally and the treatment effect of the two interventions was not significant. Also, the carry-over effect was not significant. This finding means that the interventions in the study's first phase did not affect the variables in the second phase. The effect between the study periods (comparison of the first day with the second day) was significant. The overall mean of the GRV on the second day was significantly lower than the first day (Table 3).

**Table 1:** Demographic and physiologic variables of the Mint extract and Chamomile drops groups in the first phase of the study

**Variables**	**Frequency in groups**			**p-value**
	**Mint extract**	**Chamomile drops**	**Total**	
**Age (year)**	59.63±20.80	57.49±23.30	58.59±21.90	0.665^b^
**Gender, n (%)**				
**Male**	25 (62.5)	24 (60)	49 (61.25)	0.429^c^
**Female**	15 (37.5)	16 (40)	31 (38.75)	
**Underlying diseases, n (%)**				
**Hypertension** ** n (%)**	5 (12.5)	7 (17.5)	12 (15)	0.702^c^
**Diabetes Mellitus** ** n (%)**	1 (2.5)	2 (5)	3 (3.75)	
**COPD n (%)**	0 (0)	1 (2.5)	1 (1.25)	
**More than one type n (%)**	18 (45)	17 (42.5	35 (43.75)	
**Without underlying diseases n (%)**	16 (40)	13 (325)	29 (36.25)	
**Systolic Blood** ** pressure, (mmHg)**	101.96±9.23	98.61±9.16	99.76±9.15	0.541^b^
**Heart rate** **, (bpm)**	84.20±3.18	85.09±5.60	84.68±4.08	0.232^b^
**Spontaneously respiratory rate (bpm)**	12.22±3.87	13.11±3.21	12.74±3.41	0.683^b^
**Temperature (°C)**	36.70±1.04	36.60±1.63	36.66±1.39	0.507^b^
**Gastric residual volume**	21.55±14.74	19.30±13.43	---	0.588^b^

Data are presented as n (%), or Mean±SD. SD: Standard deviation, mmHg: millimetres of mercury, bpm: beats per minute or breaths per minute, **(°C**): Degree of celsius. b independent samples T-test. c Chi-square test

**Table 2:** Comparison of the stomach residual volume before and after interventions in the Mint extract and Chamomile drops groups in the first and second phases

Phases	Groups	Times		Mean (ml)	SD (ml)	Paired Differences				t	p-value*
						Mean(ml)	SD (ml)	95% Confidence interval of the difference			
								Lower	Upper		
first phase	**Mint extract**	First time	Before (9 AM)	21.55	14.74	10.875	9.783	7.746	14.004	7.031	≤0.001
			After (12 MD)	10.68	10.07						
		Second time	Before (3 PM)	14.43	10.89	5.850	12.865	1.735	9.965	2.876	0.006
			After (6 PM)	08.58	08.19						
		Third time	Before (9 PM)	12.98	11.51	4.475	12.692	0.416	8.534	2.230	0.032
			After (24 MN)	08.50	11.83						
		Fourth time	Before (3 AM)	09.45	07.21	4.650	11.965	0.823	8.477	2.450	0.019
			After (6 AM)	04.80	10.21						
		Total times	Before	14.60	7.89	6.462	6.585	4.356	8.568	6.207	≤0.001
			After	8.13	6.31						
	**chamomile drops**	First time	Before (9 AM)	19.30	13.43	9.475	13.380	5.196	13.754	4.479	≤0.001
			After (12 MD)	9.83	11.71						
		Second time	Before (3 PM)	16.50	11.16	9.500	11.756	5.740	13.260	5.111	≤0.001
			After (6 PM)	7.00	7.40						
		Third time	Before (9 PM)	11.88	10.54	5.000	14.233	0.448	9.552	2.222	0.032
			After (24 MN)	6.88	8.29						
		Fourth time	Before (3 AM)	7.50	6.40	4.750	7.246	2.433	7.067	4.146	≤0.001
			After (6 AM)	2.75	3.57						
		Total times	Before	13.79	7.12	7.181	7.523	4.775	9.587	6.037	≤0.001
			After	6.61	4.68						
second phase	**Mint extract**	First time	Before (9 AM)	15.25	10.79	5.875	11.815	2.096	9.653	3.145	0.034
			After (12 MD)	9.37	10.13						
		Second time	Before (3 PM)	10.75	8.36	6.375	11.434	2.717	10.032	3.52	0.001
			After (6 PM)	4.37	6.42						
		Third time	Before (9 PM)	10.37	7.37	6.375	8.319	3.714	9.035	4.846	≤0.001
			After (24 MN)	4.00	4.96						
		Fourth time	Before (3 AM)	3.75	3.88	1.750	3.677	0.573	2.926	3.009	0.007
			After (6 AM)	2.00	2.95						
		Total times	Before	10.03	4.93	5.093	6.047	3.159	7.027	5.327	≤0.001
			After	4.97	4.05						
	**Chamomile drops**	First time	Before (9 AM)	15.92	11.81	4.825	14.619	0.149	9.500	2.087	0.043
			After (12 MD)	11.10	09.96						
		Second time	Before (3 PM)	13.75	12.84	5.550	13.813	1.132	9.967	2.541	0.015
			After (6 PM)	08.20	7.24						
		Third time	Before (9 PM)	10.82	11.05	5.700	9.565	2.640	8.759	3.769	0.001
			After (24 MN)	5.12	7.80						
		Fourth time	Before (3 AM)	5.35	3.80	1.850	4.335	0.463	3.236	2.699	0.010
			After (6 AM)	3.50	4.96						
		Total times	Before	11.46	7.17	4.481	7.015	2.237	6.724	4.040	≤0.001
			After	6.98	4.60						

Ml: Milliliter, AM: Morning, PM: Evening, MN: Midnight, MD: Mid-day, SD: Standard deviation. * Based on the Paired-sample T-test

**Table 3:** Distribution and parameter estimation of the various effects, mean and standard deviation of the data

Parameter	Least Squares Mean		Mean difference		T-value	Df	p-value^#^
			Mean	Standard Error			
Treatment effect^*^	Chamomile	5.800	0.022	0.663	0.033	78	0.974
	Mint	5.778					
Period effect^**^	Period 2	4.787	-2.003	0.663	-3.020	78	0.003
	Period 1	6 .790					
Carryover effect^***^	Sequence 2	12.213	1.269	2.730	0.464	78	0.643
	Sequence 1	10.944					

*Treatment effect= µChamomile-µMint. **Period effect= µPeriod2-µPeriod 1. ***Carryover effect= (µChamomile+µMint in sequence 2) - (µChamomile+µMint in sequence 1). # Significant at α<0.05

## Discussion

GRV monitoring is nursing care for patients who receive mechanical ventilation (Farsi et al., 2020 ▶; Hekmatafshar et al., 2012 ▶), which is time-consuming and causes complications (Smith, 2020 ▶). Nowadays, providers are attempting to reduce the number of GRV monitoring for patients hospitalized in ICU without harming patients (Ozen et al., 2018 ▶). The current study aimed to compare the effect of mint extract and chamomile drops on GRV reduction. In the first phase of the study, after four measurements, the mean GRV of patients before intervention in the Mint extract and Chamomile drop groups were 14.60±7.89 and 13.79±7.12 ml, respectively. In the study by Farsi et al., the GRV value before intervention in patients undergoing ventilation was 56.69±3.01 ml (Farsi et al., 2020 ▶). Lucchini et al. also reported a value of 24.4±54.2 ml for GRV before providing the intervention (Lucchini et al., 2017 ▶), which is very close to the finding of this study. This finding indicates that GRV control is critical in advanced nursing for patients undergoing ventilation.

Based on the findings, there was no significant difference between mint extract and chamomile drops in reducing GRV, and both were equally effective in reducing GRV. Also, as shown in Tables 2 and 3, using these two medicinal plants can affect GRV three hours after administration. Therefore, it seems that the interval between these two medicinal plants should be increased to more than 3 hours, or the administered dose should be reduced, which requires further investigation.

In the first phase of the present study, the mean difference of GRV before and after intervention in the Mint extract and Chamomile groups was 6.46±6.58 and 7.18±7.52 ml, respectively. In the second phase, these means were 5.09±6.04 and 4.48±7.01, respectively. Farsi et al. showed that placing patients under ventilation in supine, semi-recumbent, and right-lateral positions caused GRV to decrease by 10.38, 9.88, and 11.33 ml, respectively (Farsi et al., 2020 ▶). El-Feky et al. also showed that 15 minutes of abdominal massage could significantly reduce GRV in critically ill patients (El-Feky et al., 2020 ▶). In Rezae et al.'s study, GRV was decreased by 2.75 ml 3 hours after placing the patient at the right lateral position (Rezae et al., 2018 ▶). However, using these methods is difficult and time-consuming for nurses, and due to the heavy weight of patients, their administration may cause early burnout and neuromuscular problems.

Some studies mentioned prescribing prokinetic agents such as metoclopramide (Camilleri et al., 2013 ▶), which, similar to other medications, may cause complications. However, the results of the present study showed that mint extract and chamomile drops, which are both inexpensive and almost safe and can be found in all parts of the world, can be used to address GRV-related problems. The exact mechanism of gastric discharge by these two herbal drugs is unclear. However, some substances in these two plants help drain the stomach by relaxing sphincter pyloric and increasing the stomach and intestine peristalsis movements. Jabri et al. reported that Tunisian Chamomile (Matricaria recutita) can modulate the stomach and intestine's movement and the transmission of intestinal epithelium from water and electrolyte. Besides, having high amounts of active biological compounds can prevent gastrointestinal diseases and maintain the comfort of the intestine (Jabri et al., 2020 ▶).

Shaikh et al. stated in their article quoting Rodriguez-Fragoso et al. and Vidal et al. that "Both in pharmaceutical preparations and as a seasoning agent, Mint extract has longish credibility of safe use. In folk medicine, Mint has the credibility of successful alleviation of ailments such as parasitosis, headache, stomach cramps, flatulence and indigestion, nausea and vomiting, menstrual cramps, and dysmenorrhea. The herb was found to be of a great therapeutic benefit when employed as a counteracting agent to flu and inflammation-inducing processes of the oropharyngeal region, sinus tracts and cavities and of hepatobiliary and gastrointestinal origin”(Shaikh et al., 2014 ▶). In a study, Mokaberinejad et al. showed that mint extract did not have a significant complication or side effect (Mokaberinejad et al., 2012 ▶). Keefe et al. (2016) ▶ and Zick (2011) ▶, in their studies, showed that chamomile does not have a significant complication or adverse side effects on the human body (Keefe et al., 2016 ▶; Zick et al., 2011 ▶).

Initially, this study was designed to gavage mint extract, and chamomile drops every 3 hours. However, due to the disagreement of the physicians, the two herbal drugs were gavaged every 6 hours. Therefore, this study was limited in this regard.

Both mint extract and chamomile drops effectively reduced the GRV in patients under NGT feeding. Therefore, using mint extract or chamomile at the end of nasogastric gavage feeding is recommended in patients undergoing ventilation. Instead, continuous monitoring of GRV can be eliminated from nursing care of patients undergoing ventilation whose GRV is lower than 50 ml. However, this is a suggestion, and its implementation requires further investigation, with different doses and times.

## Conflicts of interest

The authors have declared that there is no conflict of interest.
